# Cut peony industry: the first 30 years of research and new horizons

**DOI:** 10.1093/hr/uhac079

**Published:** 2022-04-11

**Authors:** Rina Kamenetsky-Goldstein, Xiaonan Yu

**Affiliations:** 1 ARO, The Volcani Center, Institute of Plant Sciences, Israel; 2 College of Landscape Architecture, Beijing Forestry University, Beijing, China

## Abstract

Herbaceous peony is an ancient medicinal and ornamental crop, cultivated in China and Japan for thousands of years. Numerous varieties of different colors are popular garden plants in different continents and countries. In recent decades, peony has gained a new reputation as cut flowers. Only in Europe, in 30 years, trade in cut peony stems has increased 50 fold. Today, more than 25 countries produce cut peony flowers, with primary markets in Europe, Asia and the USA. This short review summarizes the contribution of research in plant physiology to the development of new technologies of peony production and flowering advancement. Despite the popularity of cut peonies, several factors still restrict their production: complicated flowering physiology, challenges in mass propagation, and postharvest handling. Further research of biochemical and molecular mechanisms, as well as breeding of new cultivars will promote the development of the peony industry and facilitate the creation of a Global Peony Chain for the successful marketing of this beautiful flower.

## Introduction

The genus *Paeonia* (Paeoniaceae) includes about 35 species, native to Asia, Europe and Western North America and renowned for their ornamental and medicinal value [1–3]. Cultivated in Chinese gardens for over a millennium, peonies were regarded as a symbol of wealth and adorned the Chinese Imperial Palace Gardens. In the 8^th^ century, peonies arrived in Japan where they became a symbol of good fortune, bravery, and honor. In the 19th century, the peonies were introduced into European and American gardens, and today it is one of the most attractive and rewarding plants in all continents [4]. According to life form, peonies are divided into tree (woody) and herbaceous species. In recent decades, herbaceous peonies are widely concerned as a new cut flower crop. The growing demand has led to new biological research and development of production technologies.

Herbaceous peonies are adapted to moderate, cold-winter climatic zones in China, Canada, the USA, and Russia. However, certain cultivars can be produced under warmer conditions in Italy, Southern France, and Southern China [5, 6]. In addition, cut peonies are also produced in New Zealand, Chile, Argentina, South Korea and Alaska.

**Figure 1:**
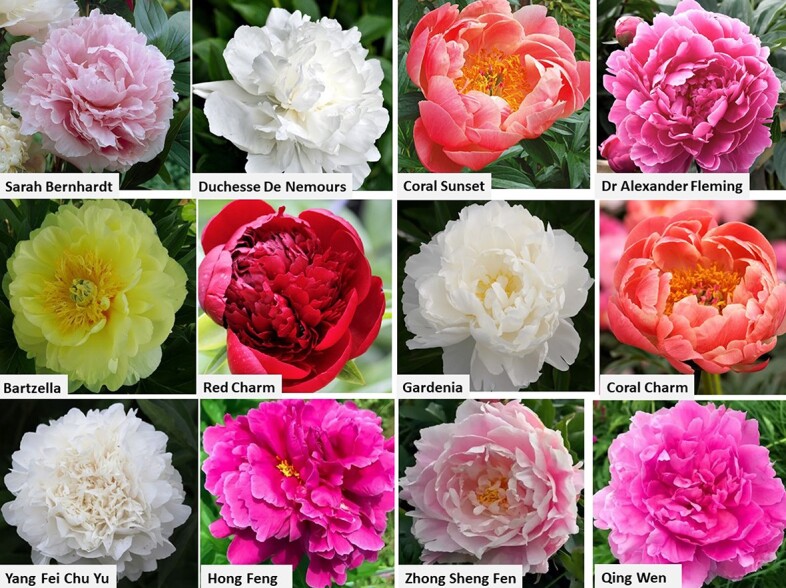
Peony varieties most popular in the Dutch and Chinese Floral Auctions (Data from Y. Kohavi; X. Yu, 2020)

The process of long-term breeding and selection of garden varieties of herbaceous peonies has resulted in great variability in flower forms and colors. According to the shape of the petal, flowers are characterized as single, Japanese, anemone, semi-double, bomb and double [7]. The double types are the most popular as cut flowers, due to their longer postharvest life. Selection criteria for cut peony flowers include round flower shape, upright, long and thick stalk, flower color, fragrance, resistance to pests and diseases, and good ability for storage and transportation. Although thousands of garden varieties are cultivated worldwide, only about 100 are used as cut flowers [8]. In Europe and the USA, the most popular cut flower varieties are “Sarah Bernhard”, “Duchesse De Nemours” and “Coral Charm”. In China, the assortment includes traditional European, as well as local varieties (Fig. 1).

**Figure 2:**
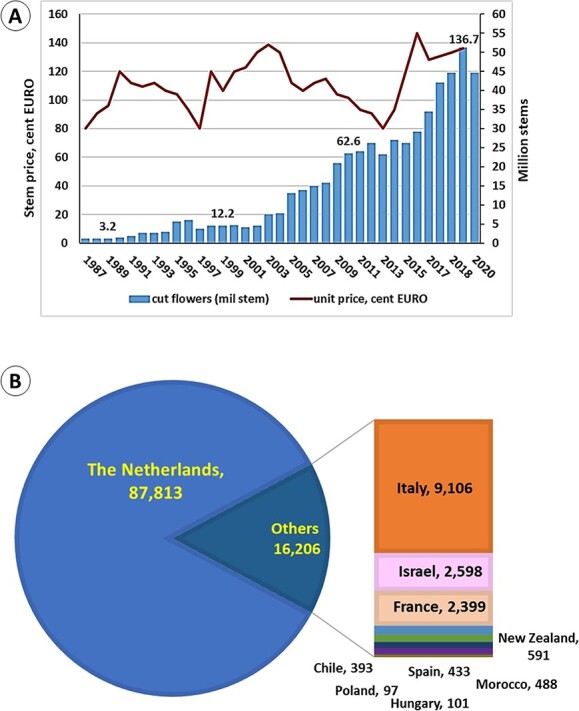
Peony trade at the Dutch Floral Auctions. A- Dynamics of peony sales and prices from 1987 to 2020; B- production of peony cut flowers (mil stems) in 2020 in Europe (Data from: Royal Flora, Y. Kohavi).

In Europe, trade in cut peony stems has increased 50 fold in 30 years, from three million stems produced at the end of the 1980’s in The Netherlands, to about 140 million stems from ca. 20 countries (Fig. 2). Interestingly, stem prices have remained stable, and today peony is one of the most expensive flowers in the flower trade. The Netherlands still leads in cut flower production, but Italy, Israel and France produce excellent flowers for the off-season trade in February–April, and therefore, production is constantly increasing.

In China, Luoyang city (Henan province) is the center of peony culture, with large living collections and gardens and dozens of annual peony exhibitions and shows. The sales volume of cut peony flowers in Luoyang increased to 15 million stems in 2021 [9]. Most of the commercial production of peony propagation material is concentrated in Heze City (Shandong province), which has become China’s largest peony planting, production, processing, export base and ornamental tourist area. In 2021, Heze produced ca. 60 million high-quality fresh cut peonies, valued at over 100 million yuan. That is 80% of the cut peony market in China [10]. In the Chinese flower market, cut peonies is are commonly sold for Chinese Mother’s Day and Internet Valentine’s Day in May, as well as for weddings. Cut peony flowers are sold mainly in Kunming, Shanghai, Beijing, Guangzhou, Shenzhen and other large domestic flower markets, and are exported to Japan, South Korea, Italy, France, the Netherlands, and Canada.

Peonies are valued in the global flower market, but in each location, they are available for only a short time in late spring and early summer. In addition, the complicated physiology of flowering and dormancy cycles and lack of advanced systems for mass propagation still restrict their production. Here we summarize the contribution of research in plant physiology to the development of new technologies of peony production and flowering advancement.

## Physiology of flowering and dormancy

### Annual cycle and its environmental regulation

**Figure 3:**
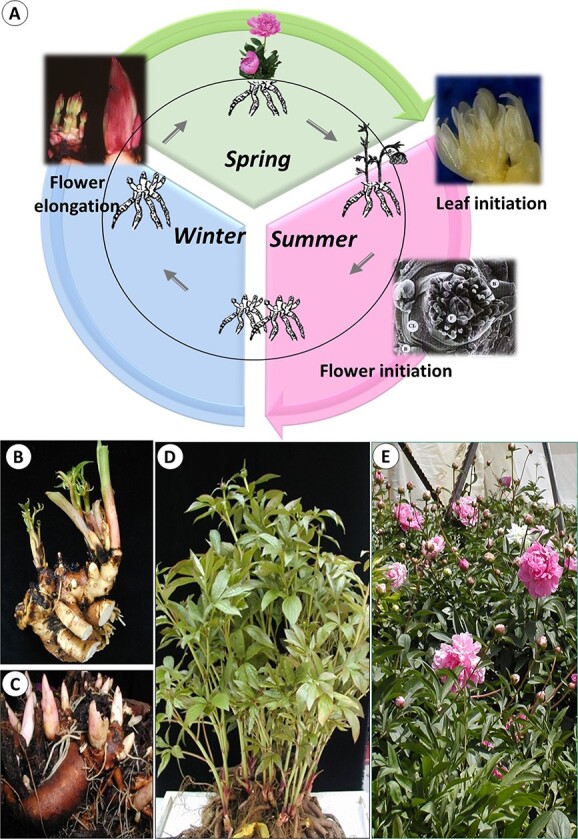
Phenological stages and annual cycle of herbaceous peony. A. Annual life cycle comprises of three main phases, regulated by low, intermediate and high temperatures. Flower initiation occurs in summer when temperatures are high, but low temperatures are required for dormancy release and successful flowering (Adapted from 11). B. Underground crown at the beginning of the growing season; C. Storage roots with renewal buds; D. Leafy stems elongate after sprouting; E. Flowering

Herbaceous peonies evolved in the climatic zone with cold winters and dry and hot summers, and their annual cycle is regulated by altering environmental conditions [11]. During winter dormancy, the underground tissues and organs carry on a series of complex physiological and biochemical reactions (Fig. 3). Renewal buds of varying sizes originate on the underground crown (Fig. 3A). In the following season, large buds that contain leaf and flower primordia, produce new flowering stems, while smaller ones remain dormant until the next season, or produce only short leafy stems (Fig. 3D). Plants flower in the spring and enter dormancy in late autumn.

In early summer, the renewal underground buds contain only vegetative meristems, while by the end of the warm period the apical meristem reaches the reproductive stage and differentiates flower parts, including bracts, calyx, petals, stamens and pistil [12**]**. After the cold period, floral stems sprout and undergo several stages on the way to anthesis (13; Fig. 3E).

Peony is a winter-dormant and deciduous plant with massive storage roots that serve as the energy supply for dormancy release and the initial growth in spring. The major storage carbohydrate in peony is starch and the basic sugars are sucrose, *myo*-inositol, fructose, and glucose [14–16]. With the resumption of growth in spring, starch concentration in the roots declines but increases again after shoot emergence and throughout flowering. The ability to accumulate starch reserves in the roots during stem growth and flowering provides an adaptive advantage in environments with a short growing season [14].

### Chilling requirements and growth conditions

During the annual cycle, environmental conditions affect all stages of dormancy, stem elongation, and flower development. In addition to air and soil temperature, plant hormones, nutritional status, air humidity, and photoperiod influence peony growth and development.

Several models have been used to assess chilling saturation in peony varieties. Storage at 2°C for 60 days is optimal for container-grown “Sarah Bernhardt” [17]. Surprisingly, the early flowering “Coral Sunset” required more chilling in comparison with the late-flowering “Sarah Bernhardt” [18]. The dormancy release of peony was affected by either constant or diurnal temperatures. Furthermore, in container-grown “Sarah Bernhardt” and “Katherine Fonteyn”, the fluctuating temperatures were found to provide higher chilling accumulation than the constant low temperature [19].

In Chinese variety “Da Fu Gui”, stem height, flower size and flowering rate were positively affected by both chilling and spraying by 200 or 500 mg/L GA_3_ [13]. However, exogenous GA_3_ does not promote flower differentiation in peonies and is not effective when is applied in summer, prior to flower induction and differentiation [20]. Uncooled peonies treated only with GA_3,_ failed to flower because of flower bud abortion [21]. In the Australian subtropics, flower production is possible when artificial chilling is combined with gibberellin application [22]. In Southern China, chilling of “Hang Baishao” at 0–4°C for four weeks and application of humic acid ensured flowering and improved its quality [6].

Growth temperatures also affect the flowering process: high temperatures promote anthesis in 50–60 days, while under continuous 22/10°C (day/night) flowering occurs after 80 days. Although “Coral Sunset”, “Monsieur Jules Elie”, “Sarah Bernhardt”, and “Karl Rosenfeld” varied in their requirements for shoot emergence and flower development, the time from emergence to flower opening was similar [23].

In warm regions, high growth temperatures have a negative effect on flower development and the quality of the cut flowers and result in complete flower abortion [17].

Malnutrition, especially lack of P and K, can also cause abortion of the flower buds in forced peony [24], while application of ABA synthesis inhibitor fluridone facilitates flowering process [25].

Therefore, under field or greenhouse conditions with large temperature variations, special techniques can prevent flower abortions. Greenhouse heating during the long and cold springs in cool regions or cooling and ventilation in warm zones will certainly improve the production of strong stems and quality flowers. The same techniques will help to prevent leaf diseases caused by high air humidity or unsuitable growth temperature [5].

The effect of photoperiod on flower formation and stem elongation in herbaceous peonies is still not clearly understood. Additional lighting for short or long periods did not affect the growth rhythm of peony plants but significantly improved the quality of the cut flowers [26]. Obviously, the effect of photoperiod and light intensity and quality should be addressed in future research for the optimization of pre- and post-harvest quality of peony flower.

### Propagation

Global expansion of cut peony would not be possible without efficient propagation systems. Due to slow seed germination, long juvenile period and offspring variation, seed propagation is employed only by breeders. The commercial companies and peony associations specialize in the vegetative propagation of garden varieties, and only some peony nurseries focus on traditional and new cut flower varieties.

Although asexual vegetative propagation is the most reliable approach, it is a rather slow method of plant reproduction [7]. The most popular technique to propagate herbaceous peonies is crown division, similar to other herbaceous plants, e.g. irises, daylilies or dahlias. The principle of this method is to divide the plant at its crown so that the separated units have shoot and root systems and are capable of producing new plants. In regions with a moderate climate, crowns with a root system are harvested in late summer or early autumn. Crown divisions include at least one bud, however 3–5 buds per division produce stronger and healthier plants.

The technique of small segments with renewal buds is similar to that of crown division. After harvest in late summer, the crown is divided into many small units. These units are planted in beds with light and well-drained mixture with constant humidity at a depth of 3–4 cm [27].

Root cuttings can be prepared for the hybrids of *P. officinalis*, *P. peregrine* and *P. tenuifolia,* which are able to form the renewal buds on storage roots [27]. Some hybrids (*e.g.* “Carol”, “Henry Bockstoce”, “Ellen Cowley”) can also be propagated using this method. However, most *Paeonia lactiflora* hybrids do not produce buds on adventitious roots.

Micropropagation of herbaceous peonies in tissue culture is based on the culture of underground buds, leaves and petioles, the induction of callus and cluster buds [28–31]. Several commercial companies are already using this biotechnology for propagating rare varieties.

### Postharvest of cut flowers

The harvest of the fresh cut flowers results in internal metabolic activity and physiological changes that diminish the flower quality. In addition, mechanical damage, inappropriate environmental conditions and microbial contaminations negatively affect the post-harvest period [32]. Proper post-harvest handling not only keeps freshness and quality of cut flowers but also can considerably enlarge market period. Therefore, both short- and long-term postharvest technologies need to be developed. Cut flower preservation technologies are based on physical, chemical and biological methods.

The methods of physical preservation of cut flowers include refrigeration, wrapping, modified atmosphere and pressure reduction [33]. The efficient way to preserve freshness after harvest is low-temperature storage. Peony stems can be stored dry or wet. Dry storage is generally used for long-term storage of peony cut flowers. The longest storage life is obtained at 0°C [34], while sub-zero storage temperatures keep cut peonies fresh for 16 weeks [35]. Dry storage for the long term accelerates starch and sucrose hydrolysis, reduces the respiration rates and expands the life of cut flowers [36]. A low-oxygen controlled atmosphere provides an additional advantage in the extension of peony vase life [37]. Wet storage of flower branches in water or fresh-keeping liquid can be used only for short period [38].

Chemical preservation of cut peonies is cost-efficient and easy to carry out. Chemical preservatives include water, sugar, fungicides, inorganic salts, organic acids, plant growth regulators and other substances. Among the various preservation solutions, sucrose and 8-hydroxyquinoline are the most basic ingredients. Nano-silver, a safe and environmentally friendly broad-spectrum nano-level inorganic antibacterial material, has been proposed for the preservation of peony flowers. Carboxymethyl chitosan (CSCM) and silicon application have been proven to have a significant effect in prolonging the life of cut stems of peony [16, 37, 39–42]. Natural extracts from Chinese medicinal plants were shown as active agents for the preservation of peony stems [43].

Taken together, the application of new preservation technologies and optimization of storage conditions can certainly prolong the post-harvest value of cut peony flowers and enlarge their potential market [37].

## Disease control

The most common peony diseases are blights and leaf spots (*Botrytis* spp.*, Cladosporium* spp., *Cercospora paeoniae, Pytophtora cactorum, Phyllosticta* spp., *Septoria paeoniae*), rots and wilts *(Fusarium* sp.*, Verticillium albo-atrum, Rhizoctonia solani, Schlerotinia schlerotiorum, Thielaviopsis basicola*), root nematodes (*Rotylenchus buxophilus, Meloidogyne* spp.) [44]. Viral diseases have been reported in Europe, the USA, Japan, China and New Zealand [45, 46].

The most important means in disease management is the diagnosis and identification of pathogens using morphological and molecular techniques. Recent recommendations take into account general disease management tools, published information on the specific biology of pathogens and long-term observations of peony diseases [47]. Botrytis bud blight can be controlled by regular spraying with a systemic fungicide and the destruction of dead foliage at the end of the season. Other fungal diseases are controlled by curative fungicide applications beginning as soon as symptoms develop.

## Production technologies

**Figure 4:**
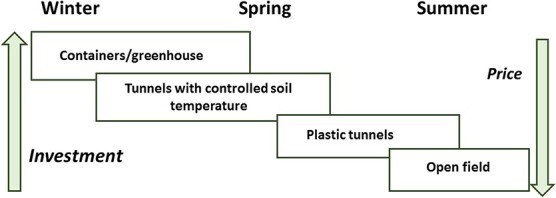
Main strategies for the production of peony cut flowers. Forcing under controlled environment in containers and tunnels results in flowering advancement. Although these technologies require higher investment, early flower production secures higher prices in the special market niches.

The majority of cut flowers worldwide are produced in open fields. This method of production is suitable for locations with cold winters when underground storage organs of peonies are naturally exposed to low temperatures and fulfill their cold requirements. Sprouting and stem elongation occur in spring, and flowering peaks in May–June in Europe, USA and Canada, and in October–November in New Zealand, Australia and South America (Fig. 4).

**Figure 5:**
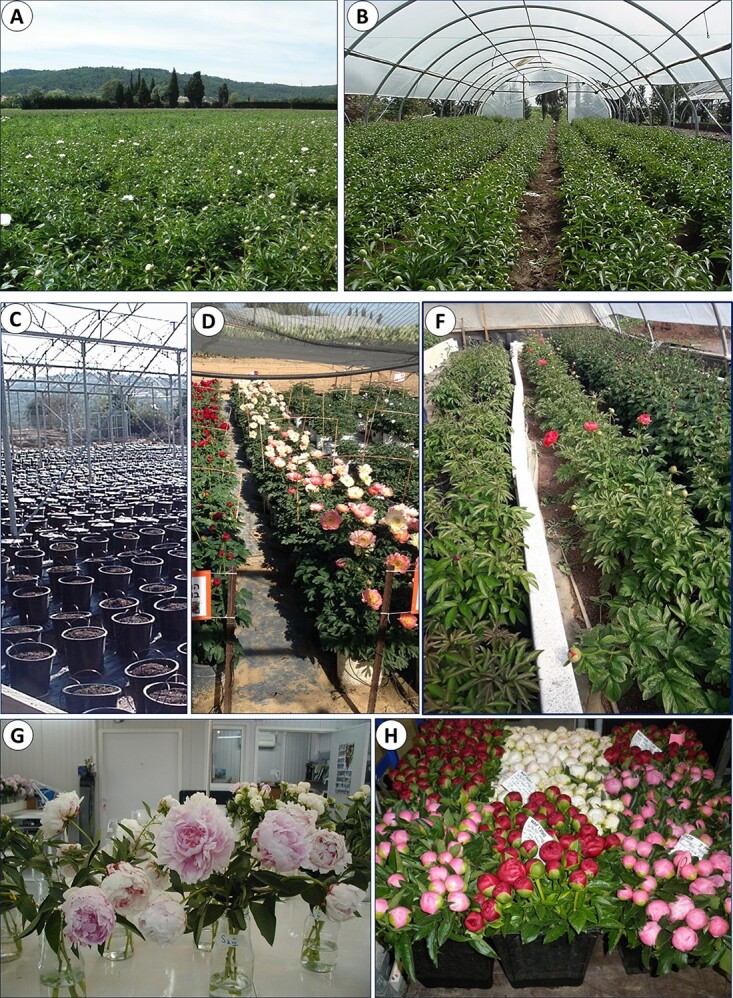
Production technologies and postharvest handling of cut peony flowers. A - Open field in South France, May 2016; B - plastic tunnel in Northern Israel, March 2014; C - pre-cooled containers with peony plants just relocated from cold storage to the greenhouse, November 2015; D - Flowering in containers, South Israel, February 2019; E- Tunnel with soil temperature control, North Israel, March 2020; G- vase life trials, Israel, 2012; H - Flower Auction in South France, May 2016 (Photo - R. Kamenetsky-Goldstein)

For flowering advancement, field-grown plants in Israel, France and Italy are exposed to natural low temperatures until they receive a certain number of chill units, predetermined for each variety (48, Figs. 4, 5). Then, the tunnels are covered with plastic, in order to raise the temperatures. This technique advances flowering for about one month than in plants grown in open fields. It requires the precise control of air temperatures during dormancy breaking, stem elongation and flower bud development. However, in covered tunnels, high temperatures might cause flower abortions or malformations of flower buds [17, 20].

Growing and forcing in containers is an efficient and sophisticated technology that requires significant investment and precise control of environmental conditions during the storage and growth stages of peony development (Figs. 4, 5). A very early crop from January to March in Israel and China and in September–October in Chile meets special market niches and gets high prices in the markets. This method is based on research on flowering biology, resource allocation and nutrition [17, 48–50]. After cold storage for 5–8 weeks and gibberellin applications, peony flowering in containers is advanced by 1–3 months in comparison with field production.

New technology of peony production in greenhouses with soil temperature control has been developed in southern France and Israel. Similar to container forcing, this green technology uses artificial cooling of soil and peony storage organs during dormancy, according to the precise temperature requirements at each physiological stage (Figs. 4, 5).

## Future outlook

### Germplasm resources and breeding research

Introduction of germplasm resources and hybridization contribute to the breeding of new cultivars with high adaptability to specific climatic conditions and disease resistance. The limiting factors in peony genetic research and breeding are high heterozygosity, poor self-compatibility and long juvenile period*.* Germplasm resources of *Paeonia* species and cultivars, the breeding strategy, and recent progress in the isolation and functional characterization of structural and regulatory genes were recently summarized by Yang et al. [51]. *P. lactiflora* is the most important species in peony breeding due to large variability in flower color and form, wide ecological adaptation, and resistance to stressful environments. Microsatellite markers developed for *P. lactiflora* will facilitate using of the germplasm sources in the breeding process [52, 53].

Sequence-related amplified polymorphism (SRAP) molecular markers were developed for the peony genotypes from the field collections in botanical gardens of the former USSR (Belarus) and USA using genome screening. Studied plants included historic cultivars of European, American and Soviet selection, interspecific hybrids and wild species [54]. For practical breeding purposes, pollen storage at 4°C and − 20°C have been developed for hybridization among cultivars and/or species with non-synchronized flowering [55]. In addition, the breeding process can be accelerated by seedling production in containers under controlled environmental conditions and a decrease of the juvenile period to 27 months [56].

### Micropropagation and biotechnology

Development of reliable *in vitro* protocols for fast multiplication of peony will certainly facilitate the rapid introduction of new varieties and disease-free material in commercial markets. Although laboratory protocols have been developed for numerous varieties [30, 57], large-scale propagation is still based on conventional methods. Contaminations, hyperhydricity and browning remain the main barriers in tissue culture establishment [30], and protocols are variety-specific.

Further, biotechnological applications and *in vitro* regeneration are important prerequisites and components of molecular research. Tissue culture provides a basic tool for many applied technologies, *e.g.* bioreactors, molecular breeding, or genetic transformation. Therefore, the establishment of the rapid propagation systems in tissue culture is of great significance for accelerating the cultivation marketing of new peony cultivars [58].

### Molecular tools and omics research

Traditional peony breeding can be greatly reinforced by the employment of modern molecular tools, *e.g.* the application of molecular markers and genomics, transcriptomic, proteomic, and metabolomic studies. Fan et al. [59] reviewed recent advances in the molecular biology of *Paeonia*, both herbaceous and tree species and cultivars. The genetic diversity of numerous germplasm collections was assessed using a range of molecular markers (AFLP, SSR, CDDP). The construction of genetic linkage maps was performed for F1 populations. In addition to -omics and miRNAs research, numerous functional genes involved in florogenesis, flower color, dormancy and stress tolerance were discovered and investigated [51]. Currently, QTL mapping and GWASs can be used to locate the genes and linkage markers associated with target traits in specific genetic populations.

**Figure 6:**
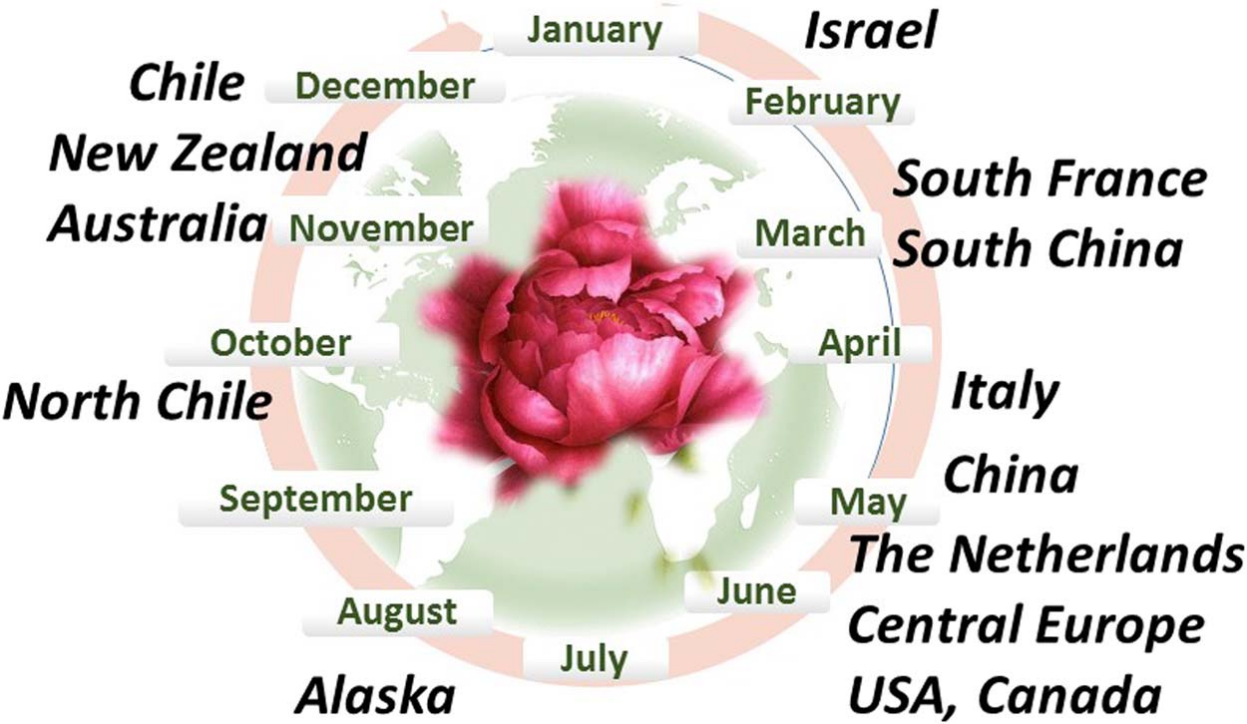
Scheme of the global production chain of cut flower peonies, using advantages of location and novel technologies. In the center: fragment of “Red Peony” by Vincent Jeannerot

Biochemical and molecular research contributes to better understanding and possible regulation of developmental traits, from stress tolerance [60, 61] to flower color [62, 63] and biosynthesis of secondary metabolites [64]. Currently, the first priority for genetic research should be introduction of disease resistance to new cultivars, and recent reports confirm possibility of this direction [65–67].

## Global Peony market chain

The development of the new industry of cut peony was successful thanks to the fruitful collaboration of many people actively involved in the production, research and marketing of this beautiful and expensive flower. National and international research programs have greatly endorsed the production of high-quality cut flowers worldwide, especially in warm-climate regions. Peony cultivation in Israel, Italy, France and South China aims at early market niche needs in February–April, in China flowering occurs from early April to mid-June. The Netherlands and Central Europe sell cut flowers in May–June, while Chile and New Zealand produce peonies in October to January. Peony production in Alaska fills the gap between Europe and USA and Southern Hemisphere. In the future, a coordinated global chain is expected to provide a constant supply of high-quality cut flowers to the international markets (Fig 6).

## Conclusions

Recent research in flowering physiology created the basis for new production technologies and flowering advancement, greatly contributing to the peony market. However, despite the popularity of cut peonies, their production is still restricted by the lack of rapid propagation systems and postharvest storage. Efficient storage and transportation techniques will allow further enlarge global peony chain and fill the gaps between various production regions. Further progress in commercial production requires breeding of new varieties, detailed studies of plant development, and improvement of production, storage, and transportation techniques. All the components of the production and marketing chain must be linked in mutually beneficial ways.
